# *Ureaplasma urealyticum* and *Mycoplasma genitalium* detection and sperm quality: A cross-sectional study in Vietnam

**DOI:** 10.18502/ijrm.v20i3.10710

**Published:** 2022-04-21

**Authors:** Minh Tam Le, Dac Nguyen Nguyen, Hoang Bach Nguyen, Viet Quynh Tram Ngo, Vu Quoc Huy Nguyen

**Affiliations:** ^1^Center for Reproductive Endocrinology and Infertility, Hue University Hospital, Hue University, Hue, Vietnam.; ^2^Department of Obstetrics and Gynecology, Hue University of Medicine and Pharmacy, Hue University, Hue, Vietnam.; ^3^Department of Microbiology, Hue University of Medicine and Pharmacy, Hue University, Hue, Vietnam.

**Keywords:** Ureaplasma urealyticum, Mycoplasma genitalium, Infertility, Spermatozoa.

## Abstract

**Background:**

*Ureaplasma urealyticum* (*U. urealyticum*) and *Mycoplasma genitalium* (*M. genitalium*) may colonize the male genital tract. However, the negative effects of these bacteria on overall sperm quality, including semen pH, sperm concentration, motility, morphology, and total sperm count remain unclear.

**Objective:**

This study aimed to determine the presence of genital *U. urealyticum* and *M. genitalium* in semen and evaluate the effect of these organisms on sperm quality.

**Materials and Methods:**

A cross-sectional study was conducted on 380 men from infertile couples at a tertiary university hospital from July 2017 to June 2018. Semen quality was analyzed according to the World Health Organization 2010 standard, and *U. urealyticum* and *M. genitalium* were detected in the semen samples using polymerase chain reaction.

**Results:**

338 men (88.9%) presented with at least one abnormal semen parameter. The detection rates of *U. urealyticum* and *M. genitalium* were 16.05% and 0.79%, respectively. There was no significant difference between the *Ureaplasma*-positive group and the *Ureaplasma*-negative group in terms of sperm characteristics. Sperm motility and sperm vitality in the *Mycoplasma-*positive group were much lower than those in the *Mycoplasma*-negative group (p = 0.02 and p < 0.001, respectively).

**Conclusion:**

The presence of *U. urealyticum* in the semen of infertile men did not affect the sperm characteristics. Although the positive rate of *M. genitalium* was low, colonization by these bacteria was more likely to negatively affect sperm quality.

## 1. Introduction

Infertility affects 8-15% of reproductive-aged couples, and male factors are the primary cause in 40% of infertility cases (1, 2). Although the cause of male infertility is often unknown, certain infertility factors can be associated with genital infections (39.6%) (3). *Mycoplasma (M.) hominis, M. genitalium, Ureaplasma (U.) parvum,* and *U. urealyticum* are pathogens that potentially play etiological roles in both genital infections and male infertility (4).Mycoplasmas and ureaplasmas, which belong to the *Mycoplasmataceae* family in the *Mollicutes* class, are widely present in humans and other mammals, birds, other vertebrates, and plants (3, 5, 6). These microorganisms are natural inhabitants of the male urethra and can contaminate semen. Moreover, they potentially play etiological roles in male infertility and genital infections (3).

*Ureaplasma spp.* and *M. genitalium* are the main causes of nonchlamydial and nongonococcal urethritis. Several reports have indicated that *Ureaplasma spp.* and *M. hominis* could lead to epididymitis; however, the impact of *Mycoplasma* infection remains unclear. Previous studies concluded that *U. urealyticum *had a negative effect on sperm quality, including semen pH, sperm concentration, mortality, morphology, and total sperm count (3). Two effects of *U. urealyticum* on sperm characteristics (inhibition of sperm motility at low pH values and increased sperm velocity at high pH values, depending on sperm metabolism) have been recently reported (7). *M. genitalium* has also been reported to be correlated with low sperm concentrations in infertile males (4). According to a meta-analysis by Pergialiotis and co-authors, bacteriospermia significantly affects sperm parameters including sperm concentration and total sperm count, and decreases rates of normal sperm morphology, vitality, and total motility. In addition, it affects semen pH and significantly impacts progressive motility (8). The authors concluded that future studies should focus on the impacts of various bacteria to corroborate their findings and enhance knowledge of the pathophysiology and treatment of male infertility (8). However, other investigators did not find any relationship between the presence of *U. urealyticum* and sperm parameters (9). To date, many studies have focused on the correlation between ureaplasmas and mycoplasmas, and sperm quality; however, the results of these studies have not been consistent.

The present study aimed to determine the relationship between *U. urealyticum* and *M. genitalium* in the semen of infertile men and their sperm quality.

## 2. Materials and Methods

### Participant population

Men were recruited from infertile couples for this cross-sectional study conducted at the Center for Reproductive Endocrinology and Infertility, Hue, Vietnam, between July 2017 and June 2018. Inclusion criteria comprised men from infertile couples, for whom semen analysis and polymerase chain reaction (PCR) tests for semen *U. urealyticum* and *M. genitalium* detection were performed. Exclusion criteria included any cases that were unable to ejaculate, those with retrograde ejaculation or sperm from cryopreservation, those who had undergone surgery, and participants with azoospermia. The participants' characteristics, including age, geography, infertility type, infertility duration, smoking status and alcohol consumption, and history of surgery on the reproductive urinary tract, were recorded.

The *Ureaplasma*-positive and *Ureaplasma*-negative groups, and the *Mycoplasma*-positive and *Mycoplasma*-negative groups were compared in terms of their sperm characteristics. The mean semen volume, pH, and sperm concentration, motility, morphology and vitality were calculated for each group.

In this cross-sectional study, the sample size was calculated through the following formula: 


$$n=Z_{\frac{\alpha }{2}}^{2}\frac{p\left(1-p\right)}{\Delta^{2}}$$

The expected prevalence of *U. urealyticum* and *M. genitalium* in the semen from infertile men was reported as 15% and 5%, respectively (4), with Z: confidence level at 95% (standard value of 1.96), p: given prevalence, and Δ: acceptable difference from prevalence (0.05). The estimated sample size was 246 cases. A total of 380 men from infertile couples were recruited for the present study.

### Semen analyses

After a physical examination, semen analyses were performed to evaluate sperm quality according to the 2010 World Health Organization (WHO) standard (10). After 3-5 days of ejaculatory abstinence, the semen samples were collected by the process of masturbation by the participants and transported to the laboratory within 30 min of ejaculation. The following parameters were evaluated according to the WHO guidelines: semen volume, concentration, progressive motility, morphology, and vitality.

Sperm motility was characterized as progressive or non-progressive by manual counting under a microscope equipped with a Primo Star (Zeiss, Jena, Germany). Sperm samples having a progressive motility of at least 32% were judged to have normal motility. The proportion of viable sperm was estimated by determining if the sperm were dead or alive using the eosin technique. Sperm morphology, including the sperm head, acrosome region, midpiece, tail and cytoplasmic droplets, was determined using contrast phase microscopy at x1000 power and using the Giemsa stain procedure.

### Conventional PCR assay for detection of *U. urealyticum* and *M. genitalium* in semen specimens

Fresh semen samples were treated according to the User-Developed Protocol of Qiagen Resources. The ^iVA^pDNA extraction Kit (Viet A Technology Corp., HCM City, Vietnam) was used for DNA extraction from the precipitated semen samples as recommended by the manufacturer. The concentration and purity of the total DNA were evaluated using a NanoDrop 2000 spectrophotometer (Thermo Scientific, MA, USA). A conventional PCR assay was performed using a forward primer (5'-AGAAGACGTTTAGCTAGAGG-3') and a reverse primer (5'-ACGACGTCCATAAGCAACT-3') that specifically targeted 540 bp of the urease gene of *U. urealyticum,* and a forward primer (5'- AGTTGATGAAACCTTAACCCCTTGG-3') and a reverse primer (5'- CCGTTGAGGGGTTTTCCA TTTTTGC-3') that specifically targeted 281 bp of the adhesin gene of *M. genitalium* (11, 12).

**Figure 1:**
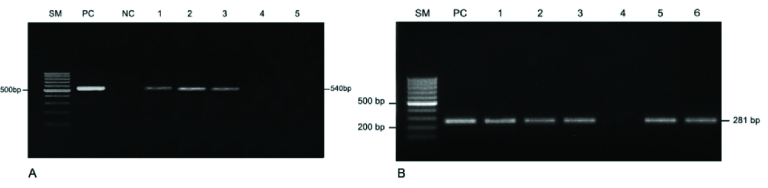
Polymerase chain reaction (PCR) amplification analysis for the detection of (A) *U. urealyticum* and (B) *M. genitalium*. PCR products were separated on a 1% agarose gel. Lane SM is a DNA size marker (GeneRuler 100 bp DNA Ladder - Thermo Fisher Scientific). In lane PC, *M. genitalium* (ATCCⓇ 33530D^TM^), and *U. urealyticum* (ATCCⓇ 29559^TM^) were used as positive controls. Lane NC is a non-template control. Lanes 3–8 are genomic DNAs from semen samples.

Five μL of extracted DNA, 0.4 μM of each primer and 12.5 μL of 2 × GoTaqⓇ Green Master Mix (Promega, Wisconsin, USA) were combined in a 25-μL total volume reaction. PCR for the detection of* U. urealyticum* was performed as follows: initial denaturation at 95 C for four min followed by 36 cycles of 95 C for 50 sec, 55 C for 50 sec, and 72 C for 60 sec. PCR for the detection of *M. genitalium* was performed as follows: initial denaturation at 95 C for five min followed by 36 cycles of 95 C for 30 sec, 65 C for 30 sec, and 72 C for 30 sec. PCR was performed in a Veriti^TM^ 96-Well Thermal Cycler (Thermo Scientific, Waltham, MA, USA). PCR products were separated through electrophoresis on a 1% agarose gel with 1 × GelRed^TM^ (Biotium, CA, USA) and digitalized with Gel Doc XR System (Bio-Rad, CA, USA). *M. genitalium* (ATCCⓇ 33530D^TM^) and *U. urealyticum* (ATCCⓇ 29559^TM^) were used as positive controls (Figure 1).

### Ethical considerations

This study was approved by the Ethics Committee of Hue University of Medicine and Pharmacy, Hue city, Vietnam (Code: H2018/103a). Informed written consent was obtained from all the participants.

### Statistical analysis

Data were presented as Mean ± SD and n (%). We categorized the characteristics as categorical variables including age ( < 35 yr, ≥ 35 yr); geography (urban, rural); type of infertiliy (primary, secondary); duration of infertility ( < 3 yr, ≥ 3 yr); body mass index ( < 23 kg/m^2^, ≥ 23 kg/m^2^), and the results of the semen parameters as defined by the WHO 2010 guidelines (10).

The Kolmogorov-Smirnov test was used to evaluate the normality of the distribution. Sperm concentration, sperm motility, and semen pH were found to be normally distributed. Therefore, standard parametric techniques (*t* tests) were used to evaluate significant differences among these factors. Because the sample volume, normal morphology, and vitality were not normally distributed, Wilcoxon rank-sum tests (Mann-Whitney U tests) were conducted. We also identified confounders that could have affected the results of the association between *U. urealyticum* and *M. genitalium* and the sperm parameters. An analysis of covariance was used when a confounding factor was recognized. The threshold for statistical significance was set at p < 0.05.

The data analyses were conducted using the Statistical Package for the Social Sciences (SPSS) (IBM Corp., Armonk, NY, USA) version 20.

## 3. Results

Of the 380 men analyzed, 61 (16.05%) were semen positive for *U. urealyticum*, and only three (0.79%) were positive for *M. genitalium*. Table I reports the demographic and baseline information of all the participants in the two groups: those who were semen PCR-positive (*U. urealyticum* positive and/or *M. genitalium* positive) and those who were semen PCR-negative. There were no significant differences between the baseline variables of the two groups.

The results of the semen parameter analysis are shown in table II. 42 (11.1%) cases had a normal sperm analysis according to the WHO 2010 guidelines, whereas 338 (88.9%) of the cases had at least one abnormal sperm parameter. All semen parameters were within the normal range (mean volume, sperm concentration, proportion of sperm with normal morphology and semen vitality), except for mean sperm motility.

Table III shows the effect of *U. urealyticum* on sperm quality. The data did not show any significant difference between the *Ureaplasma*-positive group and the *Ureaplasma*-negative group in terms of semen parameters. Patients with *Ureaplasma-*positive semen had a slightly lower rate of normal morphology sperm than did the *Ureaplasma-*negative group; however, the difference was not statistically significant (3.97 ± 2.58 vs. 4.06 ± 5.58, p = 0.50).

The relationship between the presence of *M. genitalium* and the sperm parameters is shown in table IV. Sperm motility and sperm vitality in the *Mycoplasma*-positive group were significantly lower than those in the *Mycoplasma*-negative group (sperm motility: 7.33 ± 12.70 vs. 29.11 ± 14.68, p = 0.02; and sperm vitality: 23.00 ± 39.84 vs. 71.75 ± 22.46, p < 0.001). The other indices were not significantly different between the two groups. The sperm concentration, mean sperm volume, and mean proportion of normal sperm morphology in the *Mycoplasma*-positive group were 11.33 ± 19.63, 1.50 ± 0.50 mL, and 1.00 ± 1.73%, respectively; these parameters in the *Mycoplasma*-negative group were higher than those in the positive group, at 30.70 ± 16.14, 1.73 ± 0.97 mL and 4.07 ± 5.22%, respectively, but the differences were not significant.

**Table 1:** Baseline characteristics and semen PCR results of *U. urealyticum *and* M. genitalium*


		**Semen PCR results**		**P-value**
**Characteristics**		**Positive (n = 64)**	**Negative (n = 316)**	
**Age (yr)**		35.72 ± 6.73	34.69 ± 5.53	0.25
	**< 35**	31 (48.4)	171 (54.1)	0.41
	**≥ 35**	33 (51.6)	145 (45.9)	
**Geography**				
	**Urban**	26 (40.6)	136 (43.0)	0.78
	**Rural**	38 (59.4)	180 (57.0)	
**Type of infertility**				
	**Primary**	39 (60.9)	209 (66.1)	0.47
	**Secondary**	25 (39.1)	107 (33.9)	
**Duration of infertility (yr)**				
	**< 3**	27 (42.2)	118 (37.3)	0.48
	**≥ 3**	37 (57.8)	198 (62.7)	
**History of mumps**				
	**Yes**	7 (10.9)	66 (20.9)	0.08
	**No**	57 (89.1)	250 (79.1)	
**Smoking**				
	**Yes**	26 (40.6)	114 (36.1)	0.57
	**No**	38 (59.4)	202 (63.9)	
**Alcohol consumption**				
	**Yes**	36 (56.2)	147 (46.5)	0.17
	**No**	28 (43.8)	169 (53.5)	
**BMI (kg/m^2^)**		22.85 ± 3.07	22.94 ± 3.00	0.83
	**< 23**	34 (53.1)	163 (51.6)	0.89
	**≥ 23**	30 (46.9)	153 (48.4)	
Data are presented as the Mean ± SD or number (percentage). A comparison was performed between men who were positive for *U. urealyticum* or *M. genitalium* and negative for both pathogens in the semen sample using the independent-samples *t* test. SD: Standard deviation, BMI: Body mass index, PCR: Polymerase chain reaction, Semen PCR-positive group: Positive for *U. urealyticum* or *M. genitalium* in the semen sample				

**Table 2:** Characteristics of semen parameters in the study population


**Variables**		**N (%)**	**Mean ** ± ** SD**
**Volume (mL)**		1.73 ± 0.96
	**Normal (≥ 1.5 mL) **	218 (57.4)	2.28 ± 0.95
	**Abnormal (< 1.5 mL)**	162 (42.6)	0.99 ± 0.08
**Concentration (mil/mL)**		30.54 ± 16.23
	**Normal (≥ 15 mil/mL) **	314 (82.6)	35.98 ± 11.94
	**Abnormal (< 15 mil/mL)**	66 (17.4)	4.65 ± 5.08 (8.25)*
**PR motility (%)**		28.94 ± 14.78
	**Normal (≥ 32%)**	183 (48.2)	41.39 ± 6.81
	**Abnormal (< 32%)**	197 (51.8)	17.38 ± 10.00
**Morphology (%)**		4.05 ± 5.21 (4.00)*
	**Normal (≥ 4%)**	175 (46.1)	6.64 ± 6.73 (2.00)*
	**Abnormal (< 4%)**	205 (53.9)	1.83 ± 1.05
**Vitality**		71.36 ± 22.96
	**Normal (≥ 58%)**	333 (87.6)	79.01 ± 7.63
	**Abnormal (< 58%)**	47 (12.4)	17.21 ± 22.49
**Overall **			
	**Normal **	42 (11.1)	NA
	**Abnormal **	338 (88.9)	NA
Data are presented as Mean ± SD or number (percentage). NA: Not analyzed, PR: Progressive, mil/mL: Million/milliliter, SD: Standard deviation, n (%): Sample size (percentage), *Interquartile range			

**Table 3:** Association between the presence of* U. urealyticum* and semen parameters


**Semen characteristics**	***Ureaplasma*** ***urealyticum *** **detection** * *		**Mean diff (95% CI)**	**P-value**
	**Positive (n = 61)**	**Negative (n = 319)**		
**pH**	7.16 ± 0.35	7.13 ± 0.32	0.03 (-0.06 - 0.12)	0.54
**Semen volume (ml)**	1.59 ± 0.96	1.76 ± 0.96	-0.17 (-0.44 - 0.09)	0.06
**Sperm concentration (mil/ml)**	31.16 ± 15.41	30.42 ± 16.40	0.74 (-3.72 - 5.21)	0.74
**Sperm PR motility (%)**	31.38 ± 14.51	28.47 ± 14.80	2.06 (-1.15 - 6.96)	0.16
**Normal morphology (%)**	3.97 ± 2.58	4.06 ± 5.58 (4.00)*	-0.10 (-1.53 - 1.34)	0.50
**Sperm vitality (%)**	74.69 ± 19.76	70.73 ± 23.50	3.95 (-2.35 - 10.26)	0.18
Data are presented as Mean ± standard deviation. A comparison was performed between men with positive vs. negative *U. urealyticum *detection using the independent-samples *t* test. PR: Progressive, mil/mL: Million/milliliter, CI: Confidence interval, pH: Pondus hydrogenii, diff: Difference, *Interquartile range				

**Table 4:** Association between the presence of* M. genitalium* and semen parameters


**Semen characteristics**	***Mycoplasma*** ***genitalium *** **detection** * *		**Mean diff (95% CI)**	**P-value**
	**Positive (n = 3)**	**Negative (n = 377)**		
**pH**	7.27 ± 0.46	7.13 ± 0.32	0.13 (-0.23 - 0.51)	0.47
**Semen volume (mL)**	1.50 ± 0.50	1.73 ± 0.97	-0.23 (-1.33 - 0.87)	0.92
**Sperm concentration (mil/mL)**	11.33 ± 19.63 (0-34.00)**	30.70 ± 16.14	-19.36 (-37.78 - -0.94)	0.03
**Sperm PR motility (%)**	7.33 ± 12.70 (0-22.00)**	29.11 ± 14.68	-21.78 (-38.50 - -5.06)	0.01
**Normal morphology (%)**	1.00 ± 1.73 (0-3.00)**	4.07 ± 5.22 (4.00)*	-3.07 (-9.01 - 2.87)	0.05
**Sperm vitality (%)**	23.00 ± 39.84 (0-69.00)**	71.75 ± 22.46	-48.75 (-74.49 - -23.01)	0.01
Data are presented as Mean ± standard deviation. A comparison was performed between men with positive vs. negative *M. genitalium *detection using the independent-samples *t* test. PR: Progressive, mil/mL: Million/milliliter, CI: Confidence interval, diff: Difference, *Interquartile range, **Min-Max (Interquartile range: Not applicable)				

## 4. Discussion

To date, the presence of *Mycoplasma* species is often accepted as colonization, and the impact of the presence of these organisms on male fertility remains unclear. Of the 380 men from infertile couples in this study, 61 (16.05%) were positive for *U. urealyticum*, and only three (0.79%) were positive for *M. genitalium *based on PCR testing of the semen samples. According to previous publications, *U. urealyticum* is a natural inhabitant of the male urethra and contaminates semen at varying rates (10%-42%) (13). The prevalence of *U. urealyticum* and *M. genitalium* in semen from infertile men has been reported to be 15% and 5%, respectively (4) or even higher, for example, 19.2% for *U. urealyticum*. In a recent meta-analysis of men with or without infertility, the prevalence of *U. urealyticum* was 17.53%, and the prevalence of *M. genitalium* and *M. hominis* was 11.33% and 9.68%, respectively; in particular, the rate in infertile men was higher than that in fertile men (14). The *U. urealyticum* and *M. genitalium *positive rates in our study were mostly lower than those of other studies. This may be due to the differences in the study populations. The prevalence of *U. urealyticum* infection fluctuates from 39%-48% in men aged 20-45 yr, with or without infertility and urinary symptoms (11, 15, 16). Vietnam is a developing country with limited medical resources, and infertility remains a challenge. While female genital tract infections have been widely studied (17, 18), reports of infertility-related semen infections are rare. To the best of our knowledge, this is the first epidemiological report from Vietnam and South East Asia more broadly concerning semen infection of *M. genitalium* and *U. ureaplasma*. Recognition of the impact of these pathogens and appropriate management are important to prevent sequelae on fertility.

Previous studies have illustrated that *U. urealyticum* is related to sperm quality and infertility in men (3, 4, 13). In a meta-analysis of *Ureaplasma* infection and male infertility, the former was associated with increased male infertility (p < 0.05) (19). The sperm concentration in the *U. urealyticum*-positive cohort diagnosed using urinalysis was reported to be lower than that in the *U. urealyticum*-negative cohort (p = 0.02 and p = 0.03, respectively) (20). Furthermore, a lower sperm motility rate and progressive motility were reported in the *U. urealyticum*-infected group compared to those in the uninfected group (p < 0.05) (3, 11, 16). Compared to that seen in the non-infected group, the *U. urealyticum*-infected individuals had significantly lower normal sperm morphology (9), lower pH, and lower sperm progressive motility (3). Conversely, our results found no statistically significant difference in the sperm parameters between the *U. urealyticum-*positive group and the *U. urealyticum-*negative group (p > 0.05). Our findings are in line with those of Gdoura and colleagues, who collected 120 semen samples from infertile men and found no statistically significant relationships between sperm parameters and *U. urealyticum* infection (p > 0.05) (4). Al-Sweih and co-authors also found no correlation between *U. urealyticum* infection and semen quality (volume, pH, concentration, sperm motility, and white blood cells) (p > 0.05) (9).

As demonstrated by the results above, although the correlation between *U. urealyticum* and male fertility has been widely studied, the conclusion is still controversial because of differing multifactorial aspects such as sociodemographics, economic status, sexual activity, and history of the disease. Most individuals were not aware of the infection because they did not experience any specific symptoms. The presence of *U. urealyticum* in the male genital tract is considered chronic and asymptomatic; however, *U. urealyticum* infection can cause gonadal dysfunction (13).

While a relationship between *M. genitalium* and nongonorrheal urethral infection has been reported, the impact of *M. genitalium* on male infertility remains unclear. Gdoura and co-workers reported a significantly lower sperm concentration in the *M. genitalium*-infected group than in the non-infected group (4). In examining the first-voided urine from infertile males detected with *Mycoplasma* and *Chlamydia trachomatis *(in only 1%), these organisms were found to affect semen quality (21). In contrast, other studies have concluded that *M. genitalium* does not play any role in male infertility (19). Al-Sweih and colleagues found no correlation between *M. genitalium* and semen parameters such as volume and pH, or sperm concentration, progressive motility, and total motility in infertile men; however, the white blood cell counts in infertile men with *M. genitalium* infection were higher (9). In our study, the total sperm vitality and motility in the *M. genitalium*-positive cohort were significantly lower than those in the *M. genitalium*-negative group. However, the number of *M. genitalium*-positive cases in our study was low. The correlation between *M. genitalium* infection and sperm quality in this study was not statistically significant.

Furthermore, male genitourinary infection (*Mycoplasma, Chlamydia trachomatis*) may present with increased sperm DNA fragmentation, which could result in decreased fertility (22). The present study did not determine the sperm DNA fragmentation index in the samples; therefore, it is impossible to conclude the effect of genitourinary infection (*M. genitalium, U. urealyticum*) on sperm DNA integrity in this sample. In addition, because of the low number of *M. genitalium*-positive cases, our data did not prove that *M. genitalium* had a significant negative effect on sperm quality. This could be considered a limitation of this study. Further studies with larger sample sizes should be performed to confirm the association between *M. genitalium* infection and sperm quality.

## 5. Conclusion

In conclusion, the presence of *U. urealyticum* in the semen of infertile men did not affect the sperm characteristics. Although the positive rate of *M. genitalium* was low, colonization by these bacteria was more likely to negatively affect sperm quality. Further studies with larger sample sizes that assess sperm DNA fragmentation should be conducted to determine the significant effects of the presence of *Mycoplasma* on sperm parameters.

##  Conflict of Interest

The authors declare that there is no conflict of interest.
